# Dimethyl fumarate prevents ferroptosis to attenuate acute kidney injury by acting on NRF2

**DOI:** 10.1002/ctm2.382

**Published:** 2021-05-01

**Authors:** Yunwen Yang, Fangfang Cai, Ning Zhou, Suwen Liu, Peipei Wang, Shengnan Zhang, Yue Zhang, Aihua Zhang, Zhanjun Jia, Songming Huang

**Affiliations:** ^1^ Department of Nephrology Children's Hospital of Nanjing Medical University Nanjing China; ^2^ Nanjing Key Laboratory of Pediatrics Children's Hospital of Nanjing Medical University Nanjing China; ^3^ Jiangsu Key Laboratory of Pediatrics Nanjing Medical University Nanjing China; ^4^ The State Key Laboratory of Pharmaceutical Biotechnology, College of Life Sciences Nanjing University Nanjing China

To the Editor

Epidemiological studies indicate that acute kidney injury (AKI) is still rapidly increasing every year.^1^ Currently, there is no satisfactory clinical treatment for AKI. Ferroptosis is an iron‐dependent form of regulated necrosis.^2^, ^3^ Iron metabolism and lipid peroxidation were the main inductors of ferroptosis.^2^, ^3^ This process can be inhibited by several endogenous negative regulators of ferroptosis, including GPX4 and NRF2.^3^, ^4^ Dysregulated ferroptosis has been observed in AKI induced by ischemia/reperfusion, cisplatin, and folic acid.^5^ However, there is a lack of agents targeting ferroptosis for AKI treatment. Dimethyl fumarate (DMF), an oral therapeutic small‐molecule drug has been approved by the US FDA in 2013 for therapy of multiple sclerosis and psoriasis.^6^ DMF is considered as a prodrug because it is rapidly cleaved into monomethyl fumarate (MMF) following oral administration in vivo.^7^, ^8^ Both DMF and MMF can induce NRF2 gene transcription and inhibit the degradation of NRF2, resulting in the activation of NRF2.^8^ Several studies have shown that DMF protects against tissue fibrosis and diabetic nephropathy possibly by improving inflammation and redox balance.^9^, ^10^, ^11^ However, the effect of DMF on AKI still needs further investigation.

In a cisplatin‐induced AKI mouse model (Supporting Methods), our results showed that 10 mg/kg DMF pretreatment remarkably decreased the levels of blood urea nitrogen (BUN) and serum creatinine (SCr) (Figure S1A,B; Figure 1A). Meanwhile, the substantial renal histological damage induced by cisplatin was also significantly improved after DMF treatment (Figure 1B). The Western blotting showed that the elevated kidney injury markers including KIM‐1 and NGAL were markedly decreased after DMF treatment (Figure 1C). Furthermore, the enhanced TUNEL‐positive cells induced by cisplatin were significantly decreased after DMF treatment (Figure 1D). Accordingly, the upregulation of Bax and cleaved caspase‐3 induced by cisplatin was also suppressed by DMF (Figure 1E). The results of qRT‐PCR (primer sequences are listed in Table S1) confirmed increased expression of MCP‐1, TNFα, IL‐1β, IL‐6, and COX‐2 in the kidneys of cisplatin‐treated mice, which was significantly attenuated by DMF therapy (Figure 1F). Furthermore, the ELISA assay showed that circulatory inflammatory factors including TNFα (Figure 1G) and IL‐6 (Figure 1H) were significantly reduced after DMF treatment.

**FIGURE 1:**
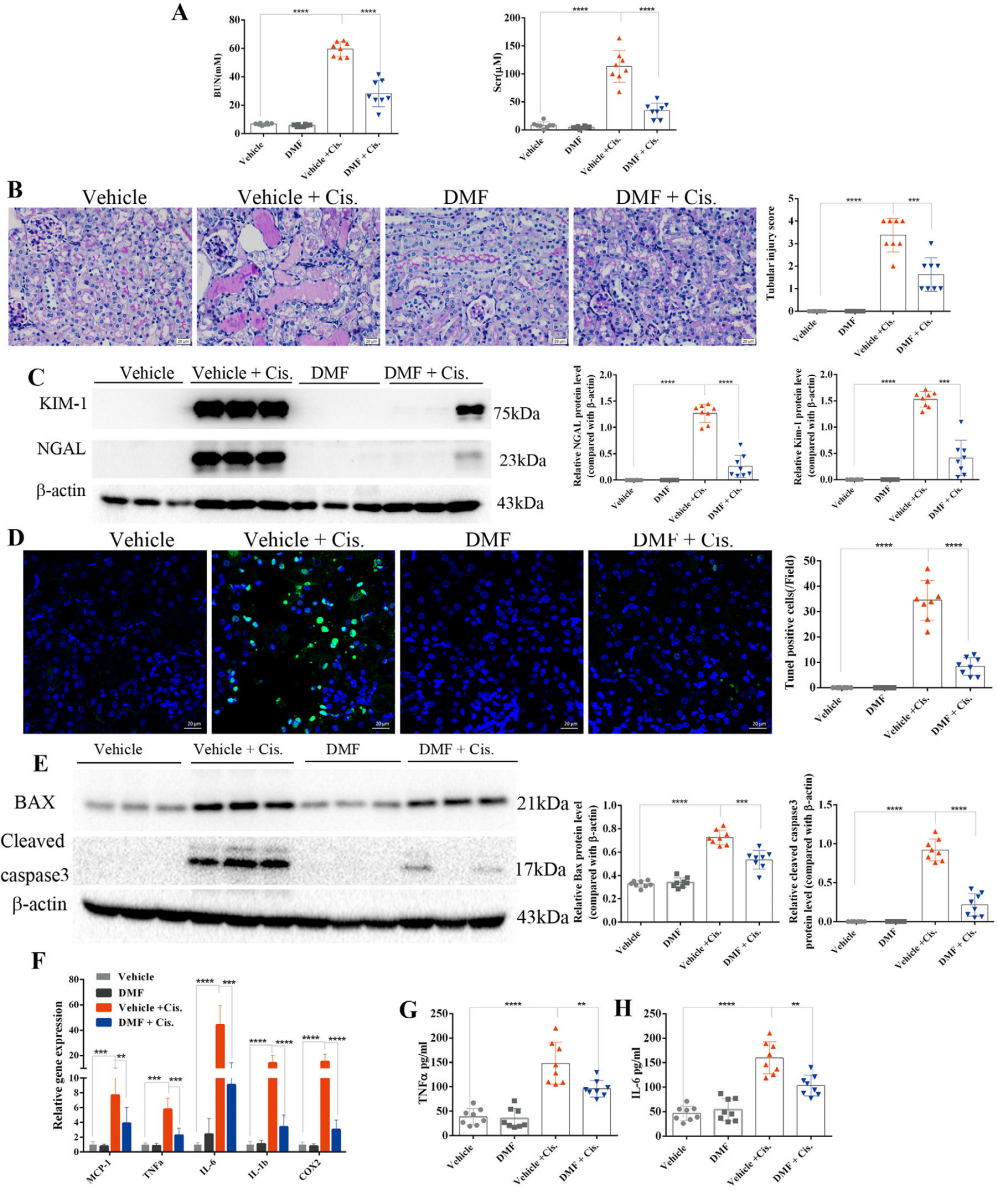
Dimethyl fumarate (DMF) treatment ameliorated cisplatin‐induced acute kidney injury in mice. (A) BUN and SCr of mice treated with 10 mg/kg DMF daily and treated with cisplatin for 72 h (*n* = 8 mice in each group). (B) PAS staining revealed that renal tubular injury induced by cisplatin was greatly reversed by DMF (10 mg/kg/day) treatment, and the renal tubular injury score was calculated (shown on the right); magnification 400×; scale bar: 20 μm. (C) Representative results of Western blotting showing that the protein levels of NGAL and KIM‐1 were significantly increased in the kidneys of cisplatin‐treated mice and were decreased after DMF (10 mg/kg/day) treatment; the images are quantified using ImageJ. (D) Representative images of TUNEL staining revealed that DMF treatment protected against cell death induced by cisplatin (magnification 400×; scale bar: 20 μm; green: TUNEL; blue: DAPI); the number of TUNEL‐positive cells in five random fields in each kidney was calculated and is shown on the right. (E) The protein levels of Bax and cleaved caspase‐3 in the kidneys of cisplatin‐treated mice with or without DMF treatment were analyzed by immunoblotting; the results of densitometry analysis performed by ImageJ are shown on the right. (F) mRNA levels of renal MCP‐1, IL‐1β, IL‐6, TNF‐α, and COX‐2 analyzed by qRT‐PCR showed that DMF (10 mg/kg/day) treatment greatly inhibited the expression of inflammatory genes. (G and H) Levels of circulating TNF‐α (G) and IL‐6 (H) were analyzed by ELISA. The results are shown as the mean ± SD of eight mice in each group. ****p < .0001, ***p < .001, **p < .01, *p < .05 (analyzed by one‐way ANOVA)

Next, genome‐wide transcriptome analysis was performed to determine possible mechanisms of DMF therapy. The RNA‐seq analysis identified more than 9000 differentially expressed genes in the vehicle versus vehicle + cisplatin groups (Supporting File S1). KEGG pathway enrichment analysis detected enriched genes associated with cisplatin‐induced cell death, mainly including apoptosis, necroptosis, and ferroptosis (Figure 2A), with higher enrichment of ferroptosis‐associated pathways than apoptosis and necroptosis (Figure 2B). A heat map of ferroptosis‐related genes showed that the expression of GPX4 was significantly decreased after cisplatin treatment and was remarkably restored by DMF therapy (Figure 2C). The results of qRT‐PCR showed that DMF treatment restored the mRNA levels of GPX4 (Figure 2D). Furthermore, DMF markedly suppressed the enhanced mRNA levels of positive ferroptosis regulators including ACSL4 (Figure 2E) and PTGS2 (Figure 2F) along with the restoration of the reduced GPX4 (Figure 2G). Interestingly, the protein levels of the NRF2 were efficiently increased after DMF treatment (Figure 2G,J; Figure S2A), which was accompanied by reduced lipid peroxidation (Figure 2I,K,M; Figure S2B), oxidative stress (Figure 2H,N), and mitochondrial damage (Figure 2L; Figure S2C).

**FIGURE 2:**
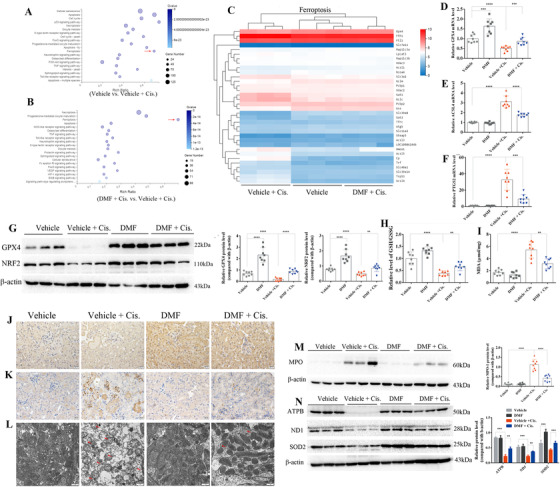
Dimethyl fumarate (DMF) treatment upregulated the nuclear translocation of NRF2 and attenuated cisplatin‐induced ferroptosis in vivo. The results of RNA‐seq analysis: (A) KEGG pathway classification and enrichment of differentially expressed genes in the vehicle versus vehicle + cisplatin groups revealed that cisplatin‐induced cell death in the kidney mainly included apoptosis, necroptosis, and ferroptosis (indicated by red arrow). (B) The results of the KEGG pathway enrichment analysis of differentially expressed genes of the cell death pathway showed that ferroptosis (indicated by red arrow), more than apoptosis and necroptosis, was the main differentially expressed pathway in the DMF + cisplatin versus vehicle + cisplatin groups. (C) Heat map of the KEGG pathway clustering of genes of the ferroptosis pathway in the vehicle, vehicle + cisplatin, and DMF + cisplatin groups. The results of qRT‐PCR showed that DMF treatment restored the mRNA levels of GPX4 (D) and decreased the mRNA levels of positive ferroptosis regulators, including ACSL4 (E) and PTGS2 (F), in the kidney of cisplatin‐treated mice. (G) The protein levels of GPX4 and NRF2 in the kidney in all groups were analyzed by Western blotting, and the quantified results are shown on the right. (H) The relative GSH/GSSG ratio (compared with the vehicle group) in the kidneys of mice treated with cisplatin (72 h) with or without DMF (10 mg/kg/day) administration. (I) Levels of MDA in the kidneys of all groups were measured by using a commercial kit. (J) Representative immunohistochemistry (IHC) staining of NRF2 in the kidneys of cisplatin‐treated mice with or without DMF treatment (magnification 400×; scale bar: 20 μm); the quantified results are shown in Figure S2A. (K) Representative IHC staining of 4‐HNE in the kidneys of all groups; the results were quantified by ImageJ and are shown in Figure S2B. (L) Representative transmission electron microscopy images of mitochondria in renal tubular cells of the renal cortex. Red arrow: damaged mitochondria (the quantified results of three mice in each group are shown in Figure S2C; cisplatin treatment for 72 h; scale bar: 1 μm). The protein levels of MPO (M), SOD2, ATPB, and ND1 (N) in the kidneys of mice were analyzed by Western blotting; the quantified results are shown on the right. The results are shown as the mean ± SD of eight mice in each group. ****p < .0001, ***p < .001, **p < .01, *p < .05 (analyzed by one‐way ANOVA)

As renal tubular epithelial cells are the most vulnerable cells in AKI,^1^ the human renal tubular epithelial cells (HK2) were used to verify the effects of DMF in vitro. DMF is rapidly cleaved into MMF in vivo; hence, we used MMF for cell culture experiments. The MMF concentrations used in this study were determined by a CCK‐8 assay (Figure S3). Interestingly, MMF markedly induced nuclear accumulation of NRF2 according to the data of Western blotting (Figure 3A) and IF staining (Figure 3B). The results of flow cytometry (Figure 3D) and LDH assay (Figure S3D) showed that the cell death induced by cisplatin was reduced after MMF treatment. Meanwhile, the MMF treatment enhanced GPX4 and decreased cleaved caspase3 determined by Western blotting (Figure 3C; Figure S3C). Accordingly, DMF inhibited cisplatin‐induced ferroptosis in HK2 cells as evidenced by lower levels of lipid peroxidation (Figure 3E; Figure S3H) and MDA (Figure S3F), and restored GSH (Figure S3E) and mitochondrial membrane potential (Figure S3I). Additionally, we also verified the protective effect of MMF against cisplatin‐induced cell death in renal mesangial cells (Figure S3L).

**FIGURE 3:**
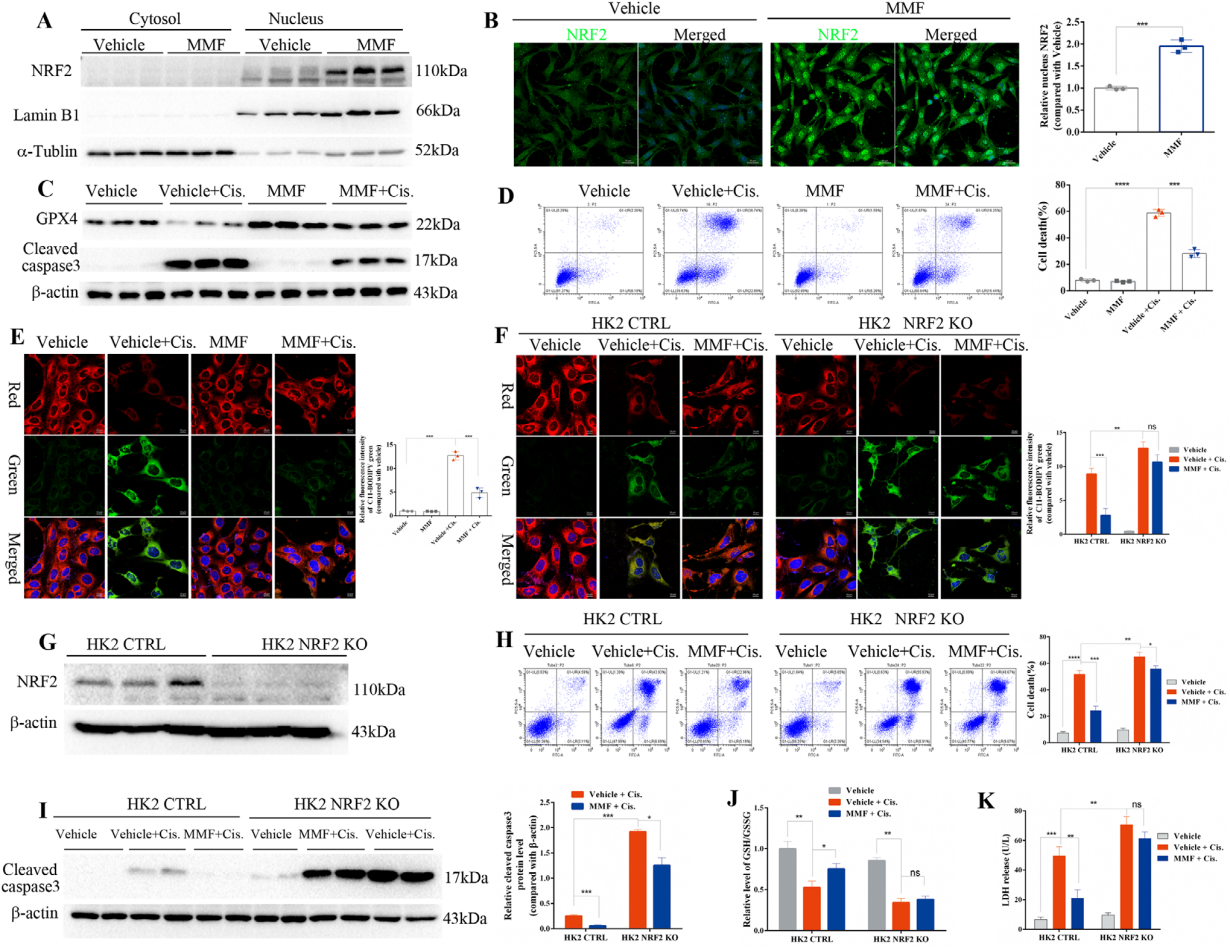
Dimethyl fumarate (DMF) promoted the activation of Nrf2 and inhibited cisplatin‐induced ferroptosis in HK2 cells. (A) Expression and subcellular localization of NRF2 were measured in HK2 cells after treatment with 10 μM MMF for 24 h by Western blotting of cellular fractions and immunofluorescence (IF) staining; the quantified results are shown on the right (B). (C) Protein levels of GPX4 and cleaved caspase‐3 in HK2 cells challenged with cisplatin with or without MMF (10 μM) were analyzed by Western blotting; the quantified results are shown in Figure S3C. (D) The death of HK2 cells induced by cisplatin was analyzed by flow cytometry; the quantified results are shown on the right. (E) Lipid peroxides were detected by staining with the fluorescent lipophilic BODIPY 581/591 C11 sensor (green: oxidized lipids; red: lipids; and blue: Hoechst; scale bar: 10 μm) analyzed by confocal fluorescence microscopy. The cells were pretreated with MMF and cultured with cisplatin (10 μg/ml) for 24 h. The graph shows the quantification of mean fluorescence intensity (MFI) ± SD of three independent experiments. (F) Representative fluorescence images of BODIPY 581/591 C11 staining of NRF2 KO and control HK2 cells (green: oxidized lipids; red: lipids; blue: Hoechst; scale bar: 10 μm). MFI of C11 was analyzed by ImageJ, and the results are shown on the right. (G) Knockout of NRF2 by the CRISPR/Cas9 strategy in HK2 cells was confirmed by Western blotting analysis and the quantified results are shown in Figure S3G. (H) The death of NRF2 KO and control HK2 cells induced by cisplatin treatment with or without MMF (10 μM) was analyzed by flow cytometry, and the quantified results are shown on the right. (I) Protein levels of cleaved caspase‐3 in NRF2 KO and control HK2 cells challenged with cisplatin with or without MMF (10 μM) were analyzed by Western blotting, and the quantified results are shown on the right. (J) The relative GSH/GSSG ratio (compared with the vehicle group) was analyzed in HK2 cells treated with cisplatin (10 mg/ml) for 24 h with or without MMF (10 μM). (K) LDH release from NRF2 KO and control HK2 cells treated with cisplatin (10 μg/ml) for 24 h; the LDH concentration was assayed in the supernatants. Three independent experiments were performed. The data are expressed as the mean ± SD. MFI: mean fluorescence intensity; ****p < .0001, ***p < .001, **p < .01, *p < .05 (two‐way ANOVA)

To determine whether NRF2 mediated the protective effect of DMF, NRF2‐knockout HK2 cells (NRF2 KO) were constructed by CRISPR/Cas9 (Figure 3G; Figure S3G), and the results showed knockout of NRF2 greatly blunted the protective effects of MMF against cisplatin‐induced ferroptosis in HK2 cells (Figure 3F–K; Figure S3J,K).

Finally, the effects of DMF on AKI were also examined in folic acid (FA)‐ and ischemia/reperfusion (IR)‐induced AKI. As shown in Figure 4A,D,G, DMF treatment markedly improved renal pathology and decreased the levels of BUN, SCr, KIM‐1, and NGAL after FA treatment for 72 h. Moreover, DMF activated NRF2 (Figure 4F) and ameliorated FA‐induced ferroptosis as evidenced by the upregulation of GPX4 (Figure 4F) and the improvement of lipid peroxidation (Figure 4B,E; Figure S4C), oxidative stress (Figure 4C), mitochondrial damage (Figure S4D), and cell death (Figure 4H). Strikingly, similar protective effects of DMF were also observed in an IR‐induced model as shown by the markedly improved renal pathology (Figure 4L) and decreased the levels of BUN, SCr, KIM‐1, and NGAL after IR for 24 h (Figure 4I,M; Figure S4E). Furthermore, IR‐induced ferroptosis was similarly inhibited by DMF (Figure 4J,K,N,O; Figure S4F).

**FIGURE 4:**
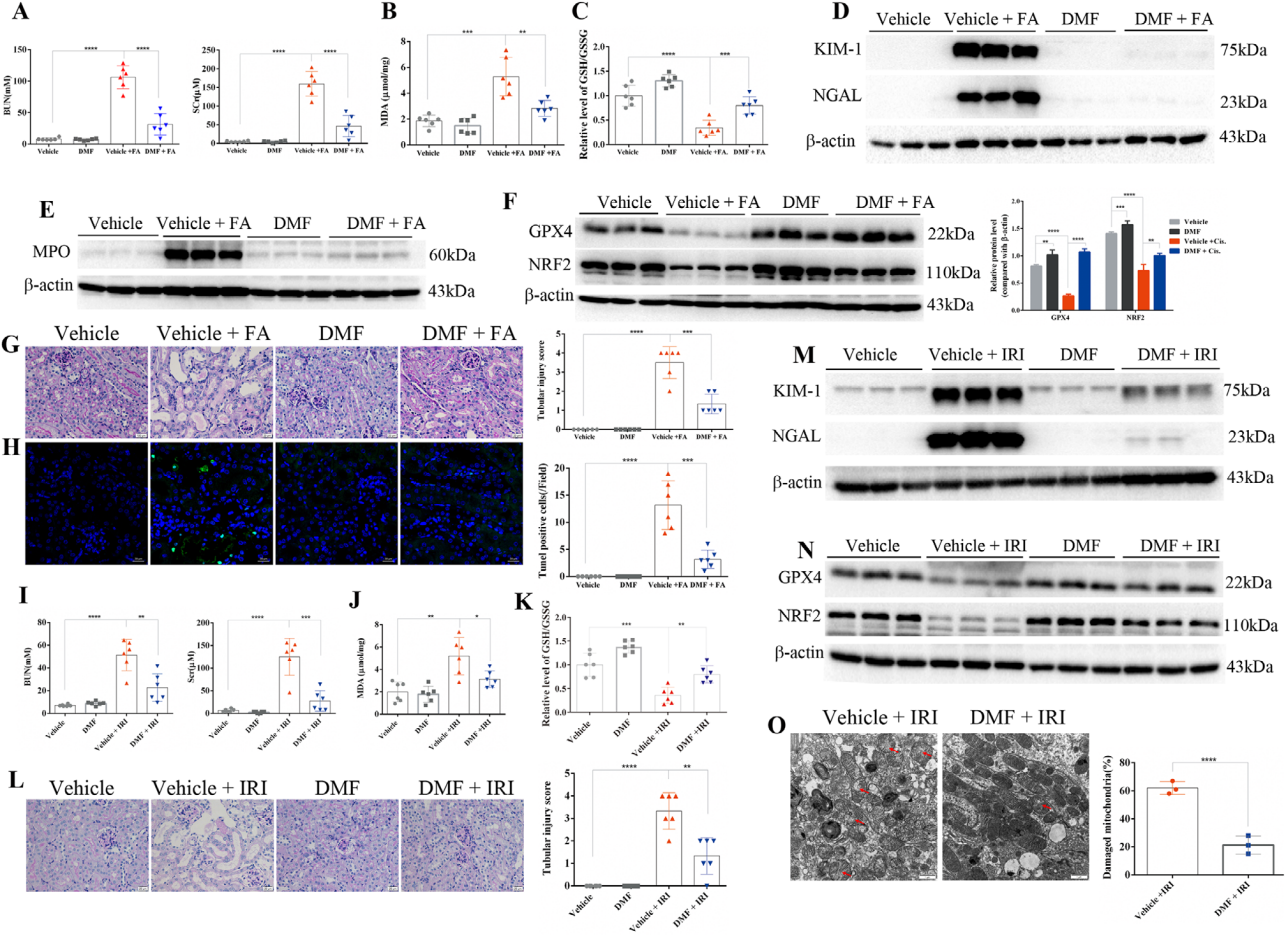
Dimethyl fumarate (DMF) treatment protected against folic acid‐ and ischemia‐reperfusion‐induced acute kidney injury. (A) BUN and SCr of FA‐treated mice with or without DMF (10 mg/kg/day) administration (*n* = 6 mice of each group). (B) DMF (10 mg/kg/day) administration decreased the upregulated levels of MDA in the kidneys of FA‐treated mice. (C) The relative GSH/GSSG ratio (compared with the vehicle group) was determined in the kidneys of mice treated with FA (72 h) with or without DMF (10 mg/kg/day) administration. The protein levels of KIM‐1, NGAL (D), MPO (E), GPX4, and NRF2 (F) in the kidneys of FA‐treated mice with or without DMF administration were analyzed by immunoblotting, and the results of densitometry analysis performed by ImageJ are shown on the right; Figure S4A,B. (G) Representative PAS staining images (scale bar: 20 μm) of the kidneys in all groups. Tubular injury scores were analyzed (right). (H) Representative images of TUNEL staining revealed that DMF treatment protected against cell death induced by FA (scale bar: 20 μm; green: TUNEL; blue: DAPI); the number of TUNEL‐positive cells in five random fields from each kidney are shown on the right. (I) BUN and SCr of ischemia‐reperfusion injury (IRI) mice with or without DMF (10 mg/kg/day) administration (*n* = 6 mice in each group). (J) DMF (10 mg/kg/day) administration decreased the upregulated levels of MDA in the kidneys of IRI mice. (K) The relative GSH/GSSG ratio (compared with the vehicle group) was analyzed in the kidneys of IRI mice (24 h) with or without DMF (10 mg/kg/day) administration. (L) Representative PAS staining images (scale bar: 20 μm) of the kidneys in all groups. Tubular injury scores were analyzed (right). The protein levels of KIM‐1, NGAL (M), GPX4, and NRF2 (N) in the kidneys of IRI mice with or without DMF treatment were analyzed by immunoblotting, and the results of densitometry analysis performed by ImageJ are shown in Figure S4E,F. (O) Representative electron microscopy images and quantification of damaged mitochondria in renal tubular cells (the quantified results are shown as the mean ± SD of three mice in each group; IRI for 24 h; scale bar: 1 μm). The quantified results are shown as the mean ± SD of six mice in each group. ****p < .0001, ***p < .001, **p < .01, *p < .05 (one‐way ANOVA). FA, folic acid; IRI, ischemia‐reperfusion injury

Apoptosis, necroptosis, and ferroptosis are the main pathological features of cisplatin‐ and IR‐induced AKI.^5^ However, a study reported that ferroptosis not necroptosis was the primary cause of FA‐induced AKI.^12^ Our results demonstrated that DMF prevented ferroptosis and ameliorated AKI possibly by acting on NRF2 and anti‐peroxidation, suggesting the clinical potential of DMF for AKI therapy.

## FUNDING INFORMATION

National Key Research and Development Program, Grant number: 2016YFC0906103; National Natural Science Foundation of China, Grant number: 81700642; Natural Science Foundation of Jiangsu Province, Grant number: BK20170150; China Postdoctoral Science Foundation, Grant number: 2018M640505; Jiangsu Postdoctoral Science Foundation, Grant number: 2018K042A

## CONFLICT OF INTEREST

The authors declare that there is no conflict of interest.

## AUTHOR CONTRIBUTIONS

Yunwen Yang, Fangfang Cai, Suwen Liu, and Ning Zhou performed the experiments and prepared the figures. Songming Huang, Zhanjun Jia, and Yunwen Yang designed the experiments, analyzed the data, and wrote the main text of the manuscript. Peipei Wang, Shengnan Zhang, Aihua Zhang, and Yue Zhang offered the assistance with the manuscript preparation, and all the authors reviewed the manuscript.

## DATA AVAILABILITY STATEMENT

The data that support the findings of this study are available from the corresponding authors upon reasonable request.

## ETHICS APPROVAL AND CONSENT TO PARTICIPATE

All animal procedures of this study were approved by the Institutional Animal Care and Use Committee of Nanjing Medical University (registration number: IACUC 14030112‐2).

## Supporting information

Figure S1

Figure S2

Figure S3

Figure S4

Supporting Materials

Supporting Information File S1
