# Supplementary material for: Dimethyl fumarate prevents ferroptosis to attenuate acute kidney injury by acting on NRF2
Source: Clin Transl Med. 2021 May 1;11(4):e382. doi: 10.1002/ctm2.382 (PMC8087913; doi:10.1002/ctm2.382)
Supplement: Supplementary file 1 — Figure S1 [file CTM2-11-e382-s001.docx]

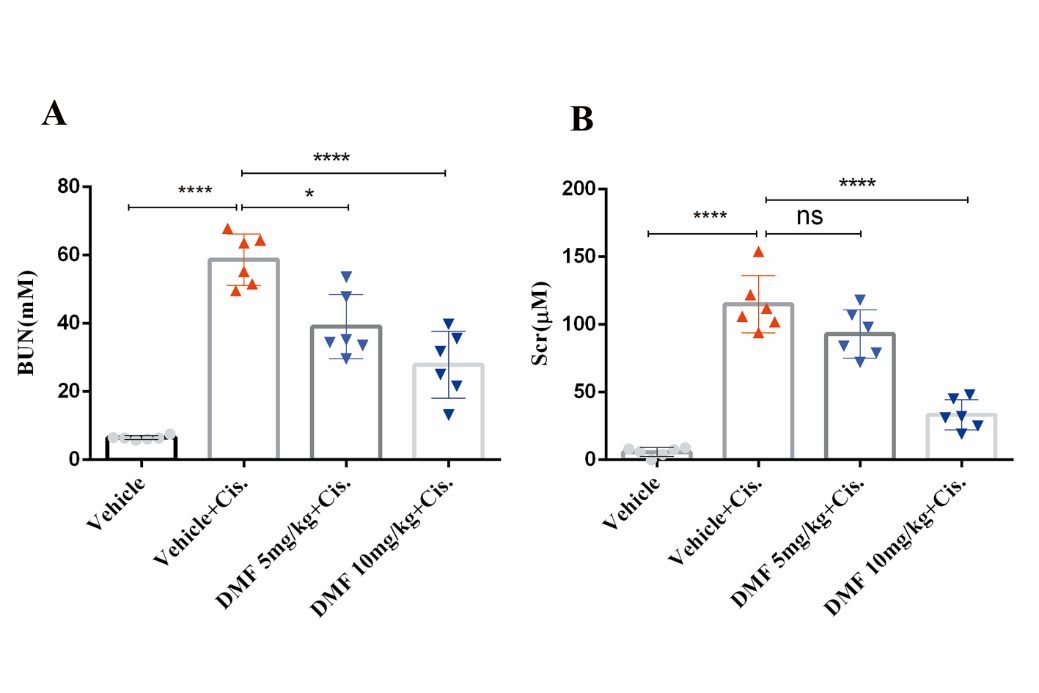


**Supplementary Figure S1**. **The dose response of DMF on renal function.** (A) Blood urea nitrogen (BUN) and (B) serum creatinine (SCr) of mice treated with various doses of DMF by gavage and treated with cisplatin for 72 h. The data are presented as the mean ± S.D. of each mouse (n = 6 mice in each group). *****P<0.0001, ***P < 0.001, **P<0.01, *P<0.05* (analyzed by one-way ANOVA)*.* ns: not significant
