# Supplementary material for: Dimethyl fumarate prevents ferroptosis to attenuate acute kidney injury by acting on NRF2
Source: Clin Transl Med. 2021 May 1;11(4):e382. doi: 10.1002/ctm2.382 (PMC8087913; doi:10.1002/ctm2.382)
Supplement: Supplementary file 2 — Figure S2 [file CTM2-11-e382-s005.docx]

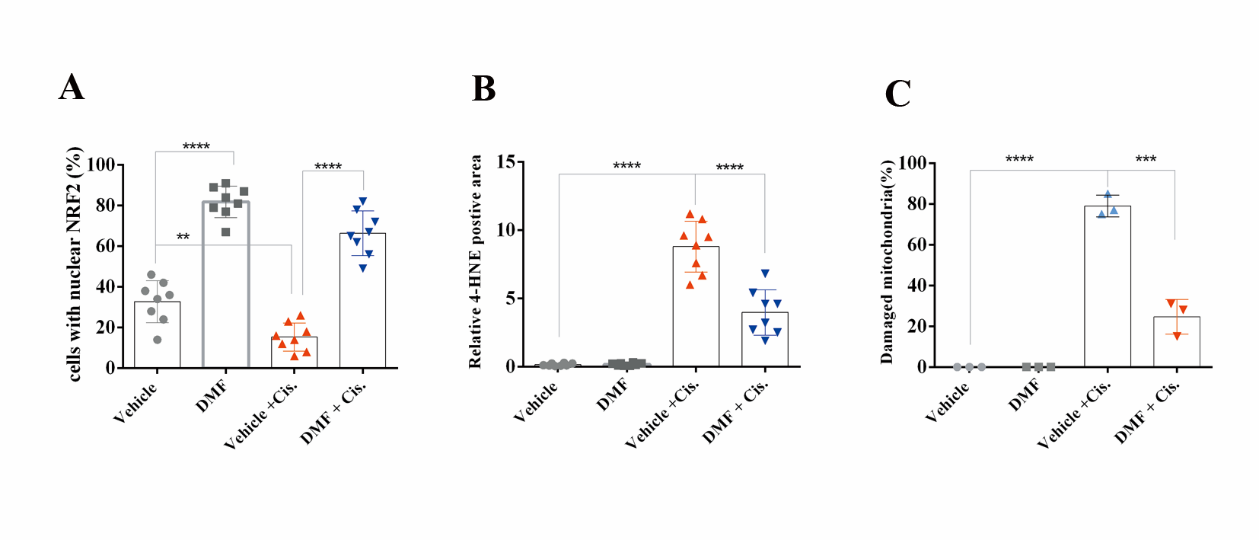


**Supplementary Figure S2.** **The quantified results of Figure 2.** (A) The quantified results of IHC staining of NRF2 (Figure 2J) performed by ImageJ. The results are shown as the mean ± S.D. of 8 mice in each group. *****P<0.0001, **P<0.01 (*one way ANOVA*).* (B) The quantified results of IHC staining of 4-HNE (Figure 2K) performed by ImageJ. The results are shown as the mean ± S.D. of 8 mice in each group. *****P<0.0001(*one-way ANOVA*).* (C) The quantified results of damaged mitochondria (Figure 2L) of 3 mice in each group, *****P<0.0001, ***P<0.001 (*one-way ANOVA*)*. Cis. : Cisplatin.
