# Supplementary material for: Dimethyl fumarate prevents ferroptosis to attenuate acute kidney injury by acting on NRF2
Source: Clin Transl Med. 2021 May 1;11(4):e382. doi: 10.1002/ctm2.382 (PMC8087913; doi:10.1002/ctm2.382)
Supplement: Supplementary file 3 — Figure S3 [file CTM2-11-e382-s006.docx]

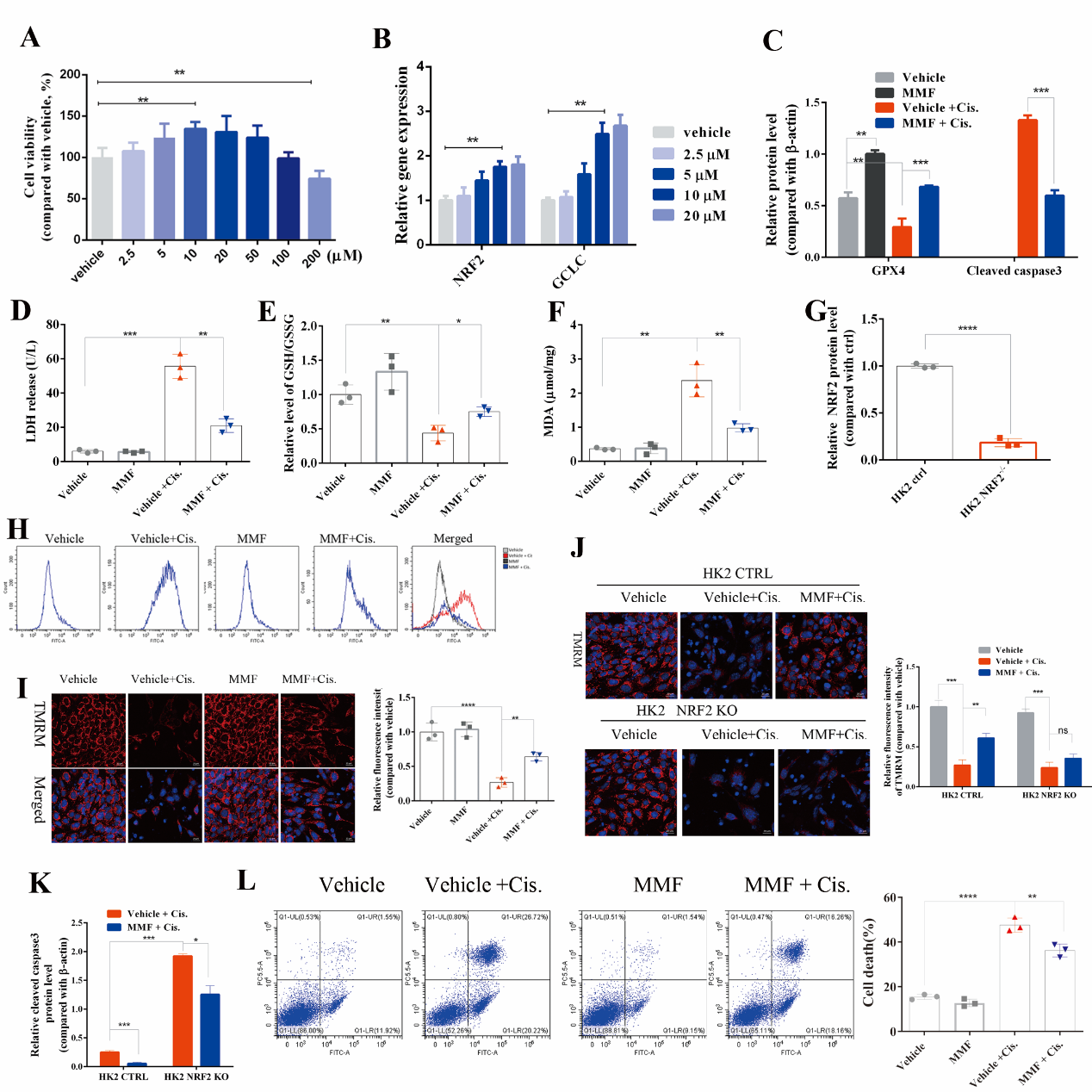


**Supplementary Figure S3**. **DMF promoted the activation of Nrf2 and inhibited cisplatin-induced cell death in HK2 cells and mesangial cells**. (A) Viability of HK2 cells after treatment with MMF concentrations from 2.5 μM to 200 μM for 24 h was analyzed by CCK-8 assay. (B) The mRNA levels of NRF2 and one of its target genes, GCLC, after treatment of HK2 cells with MMF (2.5, 5, 10, and 20 μM) were analyzed by qRT-PCR. (C) The results of densitometry analysis (Figure 4C) performed by ImageJ. (D) MMF treatment decreased LDH release from HK2 cells treated with cisplatin (10 μg/ml) for 24 h; the supernatants were collected at indicated time points and assayed for LDH concentration. (E) The relative GSH/GSSG ratio (compared with the control group) and the levels of MDA (F) in HK2 cells treated with cisplatin with or without MMF (10 μM). (G) The quantified results of NRF2 protein levels (Figure 3G). (H) Representative FACS analysis of HK2 cells stained by BODIPY 581/591 C11 sensor (Green). The cells were pretreated with MMF and cultured with cisplatin (10 μg/ml) for 24 h. (I) Representative fluorescence images of mitochondrial membrane potential (TMRM) in HK2 cells (red: TMRM; blue: Hoechst; scale bar: 20 μm) and MFI of TMRM analyzed by ImageJ on the right. Three independent experiments were performed in the cells. (J) Representative fluorescence images of TMRM in NRF2 KO and control HK2 cells (red: TMRM; blue: Hoechst; scale bar: 20 μm); the analysis of MFI of TMRM by ImageJ is shown on the below. (K) The results of densitometry analysis (Figure 4I) performed by ImageJ. (L) The death of mesangial cells induced by cisplatin treatment with or without MMF (10 μM) was analyzed by flow cytometry, and the quantified results are shown on the right. Three independent experiments were performed. The data are expressed as the mean ± S.D. *****P<0.0001, ***P<0.001, **P<0.01, *P < 0.05* (two-way ANOVA)*, ns:* not significant.
