# Supplementary material for: Dimethyl fumarate prevents ferroptosis to attenuate acute kidney injury by acting on NRF2
Source: Clin Transl Med. 2021 May 1;11(4):e382. doi: 10.1002/ctm2.382 (PMC8087913; doi:10.1002/ctm2.382)
Supplement: Supplementary file 4 — Figure S4 [file CTM2-11-e382-s002.docx]

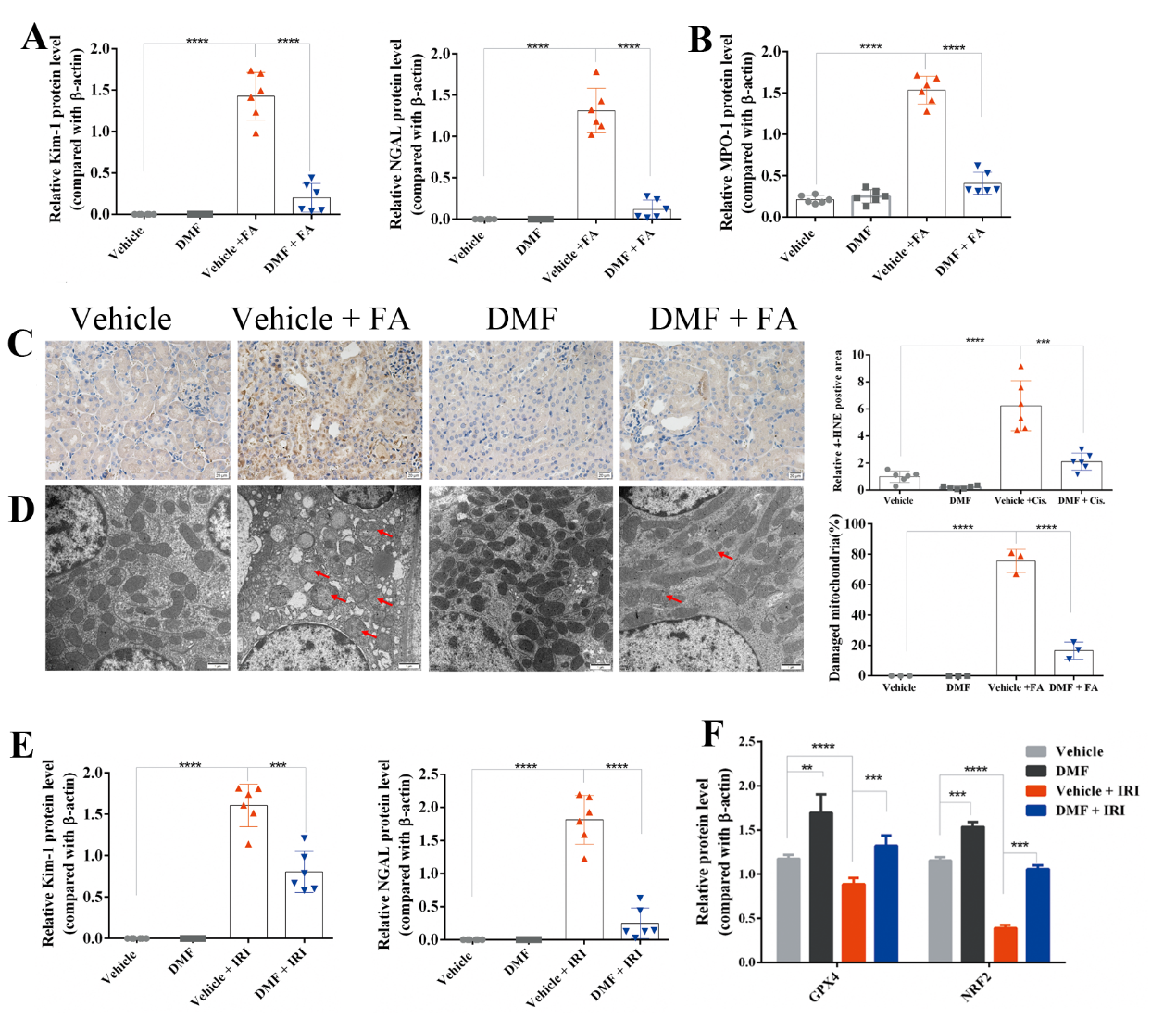


**Supplementary Figure S4**. **DMF treatment protected against folic acid induced lipid peroxidation and the quantified results of Western blot of Figure 4N.** (A) The results of densitometry analysis of Kim-1 and NGAL (Figure 4D) performed by ImageJ. (B) The results of densitometry analysis of MPO (Figure 4E) performed by ImageJ. (C) Representative IHC staining of 4-HNE in the kidneys in all groups; the results were quantified by ImageJ (right). (D) Representative electron microscopy images and quantification of damaged mitochondria in renal tubular cells (the quantified results are shown as the mean ± S.D. of 3 mice in each group; FA treatment for 72 h; scale bar: 1 μm). The results of densitometry analysis of Kim-1 and NGAL (Figure 4M) performed by Image J. (F) the results of densitometry analysis of GPX4 and NRF2 (Figure 4N) performed by ImageJ. The quantified results are shown as the mean ± S.D. of 6 mice in each group. FA: folic acid; IRI: ischemia-reperfusion injury; *****P<0.0001, ***P < 0.001, **P<0.01, *P<0.05 (*two-way ANOVA*)*.
