# Supplementary material for: Dimethyl fumarate prevents ferroptosis to attenuate acute kidney injury by acting on NRF2
Source: Clin Transl Med. 2021 May 1;11(4):e382. doi: 10.1002/ctm2.382 (PMC8087913; doi:10.1002/ctm2.382)
Supplement: Supplementary file 6 — Supporting Information File S1 [file CTM2-11-e382-s004.docx]

**Materials and methods**

**Mouse models of AKI**

Eight- to ten-week-old male C57BL/6J mice were purchased from the Laboratory Animal Center of Nanjing Medical University (Nanjing, China). Mice were maintained at 21.0 °C and 55.0 ± 5.0% relative humidity with a 12:12-h light/dark cycle and free access to food and water in a standard SPF animal room. Initially, the effects of DMF (MedChemExpress, HY-17363) were examined in a cisplatin-induced acute kidney injury mouse model. Mice were randomly divided into 4 groups: vehicle (0.5% methyl cellulose) group (vehicle, n=8), DMF gavage group (DMF, n=8), cisplatin-treated group (vehicle + cisplatin, n=8), and cisplatin plus DMF group (DMF + cisplatin, n=8). The mice of the cisplatin and DMF + cisplatin groups received a single intraperitoneal (i.p.) injection of cisplatin (dissolved in saline, 25 mg/kg), and mice in the vehicle and DMF groups received an equal volume of saline. Mice in the DMF treatment groups were gavaged with 10 mg/kg/d DMF dissolved in 0.5% methyl cellulose once daily 72 h before i.p. injection of cisplatin, and gavage with DMF was continued once daily until the mice were sacrificed. The mice in the other groups received an equal volume of vehicle (0.5% methyl cellulose) once daily. Additionally, the therapeutic effects of DMF were examined in a folic acid (FA)-induced AKI model; mice were randomly divided into four groups (n=6 per group): vehicle group (vehicle), DMF-treated group (DMF), FA-treated vehicle group (vehicle + FA), and FA plus DMF-treated group (DMF + FA). Mice in the DMF treatment groups were gavaged with 10 mg/kg/d DMF dissolved in 0.5% methyl cellulose once daily 72 h before i.p. injection of 250 mg/kg FA (F7876, Sigma, St. Louis, MO) in 0.15 M NaHCO_3_, and gavage with DMF was continued once daily until the mice were sacrificed. Mice of all groups were sacrificed 72 h after i.p. injection of cisplatin or FA. In the case of an ischemia-reperfusion injury (IRI)-induced AKI model, mice were randomly divided into four groups (n=6 per group): vehicle group (vehicle), DMF-treated group (DMF), IRI group (vehicle + IRI), and IRI-induced DMF-treated group (DMF + IRI). Mice in the DMF treatment groups were gavaged with 10 mg/kg/d DMF once daily 72 h before I/R surgery. IRI surgery was performed as described in our previous study ^[^[^36^](#_ENREF_36)^]^. Briefly, mice were anesthetized with isoflurane, and bilateral renal artery clipping was performed for 35 min using microaneurysm clamps (Aesculap, Tuttlingen, Germany). Mice in the control group underwent the same procedure without clamping. After 24 h, the mice of all groups were euthanized. Serum and kidneys were collected and stored at −80 °C for subsequent analysis. Kidney tissues were fixed in 4% paraformaldehyde for histological analysis. Serum creatinine (SCr) and blood urea nitrogen (BUN) were assayed using an automatic biochemical analyzer at the Children’s Hospital of Nanjing Medical University. All animal procedures of this study were approved by the Institutional Animal Care and Use Committee of Nanjing Medical University (registration number: IACUC 14030112-2).

**Cell culture and treatment**

The human proximal tubule epithelial cell line (HK2) was obtained from American Type Culture Collection (ATCC, Manassas, VA). HK2 cells were cultured in DMEM/F-12 medium (Gibco, 319-075-CL) supplemented with 10% fetal bovine serum (FBS, Gibco, 26170035), penicillin (100 U/mL), and streptomycin (100 µg/mL) at 37 °C in a humidified incubator in the presence of 5% CO_2_. FBS was removed from the medium when the cells reached 70% confluence, and the cells were pretreated with MMF (MedChemExpress, HY-103252, dissolved in DMSO) for 1 h. Then, 10 μg/mL cisplatin was added to HK2 cells for 24 h. CRISPR/Cas9 was used to knock out NRF2 in HK2 cells, and the sgRNAs targeting NRF2 were cloned into the pSpCas9(BB)-2A-Puro (PX459) v2.0 vector, which was a gift from Feng Zhang (Addgene plasmid # 62988), as described in a previous study ^[^[^37^](#_ENREF_37)^]^. The sequences are listed in Supplementary Table S1. The sequenced CRISPR/Cas9 plasmids targeting NRF2 were transfected into HK2 cells with PolyJet™ DNA transfection reagent (SignaGen, SL100688), and the positive cells were selected with puromycin (2 μg/mL) for three days prior to clonal expansion. HK2 cells transfected with PX459 were used as a control.

**Quantitative real-time PCR (qRT-PCR)**

TRIzol (TAKARA, Dalian, China; 9108) was used to extract total RNA from the kidney tissues or cultured cells according to the manufacturer’s instructions. One microgram of RNA sample was reverse transcribed using a reverse transcriptase M-MLV kit (TAKARA, 2641A). The primers (Supplementary Table S1) were designed and synthesized by Tsingke Biotech (Nanjing, China). Messenger RNA (mRNA) expression was measured by qRT-PCR by a QuantStudio 3 real-time PCR system (Applied Biosystems, Foster City, CA, USA) using SYBR Green master mix (Vazyme, Nanjing, China; q111-02/03). Cycling conditions of qRT-PCR were 95 °C for 10 min followed by 40 cycles at 95 °C for 15 s and 60 °C for 1 min. The relative threshold cycle values (ΔCt) were used to analyze and calculate the relative levels of mRNA expression normalized to β-actin that was used as an internal control; then, fold changes were estimated using the 2^−ΔΔCt^ method as described previously ^[^[^38^](#_ENREF_38)^]^ to determine the relative mRNA levels.

**Western blotting**

Western blot analysis of the kidney tissue and cell extracts was performed as described in our previous study ^[^[^39^](#_ENREF_39)^]^. Briefly, 30 μg of total protein from each sample was used for western blot analysis performed according to a routine protocol. Primary antibodies against KIM-1 (R&D Systems, AF1817, 1:1,000), NGAL (Abcam; ab63929, 1:1,000), BAX (Proteintech; 50599-2-Ig, 1:1,000), cleaved caspase-3 (Cell Signaling Technology; 9664, 1:1,000), ATPB (Proteintech; 17247-1-AP, 1:1,000), SOD2 (Proteintech; 24127-1-AP, 1:1,000), ND1 (Proteintech; 19703-1-AP, 1:1,000), NRF2 (Proteintech; 16396-1-AP, 1:1,000), myeloperoxidase (MPO) (R&D Systems; AF3667, 1:1,000), GPX4 (Proteintech; 14432-1-AP, 1:1,000), β-actin (Proteintech; 66009-1-Ig, 1:1,000), and lamin B1 (Proteintech; 12987-1-AP, 1:1,000) were diluted in 5% skim milk prepared in TBST buffer (Tris-buffered saline containing 0.1% Tween 20). Peroxidase-conjugated goat anti-rabbit (Beyotime; A0208) or anti-mouse (Beyotime; A0216) secondary antibodies were diluted 1:1,000. Immunoblotting images were captured using an enhanced chemiluminescence detection system (Bio-Rad, Hercules, CA, USA), and densitometric analysis of immunoblotting bands was performed by using ImageJ (Wayne Rasband, National Institutes of Health, USA).

**Histological analysis**

The kidney tissues were fixed in 4% paraformaldehyde overnight, embedded in paraffin wax, and serially sectioned at 4 μm thickness for staining. Periodic acid-Schiff (PAS) staining was performed to analyze histological changes in the kidney tissue, and the images were captured with an Olympus BX51 microscope (Olympus, Center Valley, PA). A score from 0 to 4 was assigned based on pathological damage of the tissue by calculating the percentage of damaged renal tubules characterized by cell lysis, tubular dilation, loss of brush border, and cast formation: 0, no abnormalities; 1+, <25%; 2+, 25–50%; 3+, 50–75%; and 4+, >75% ^[^[^40^](#_ENREF_40)^,^ [^41^](#_ENREF_41)^]^.

**Immunohistochemistry (IHC) and immunofluorescence (IF) staining**

IHC staining was performed as described in our previous study ^[^[^39^](#_ENREF_39)^]^. Briefly, the kidney sections were incubated with primary antibody against NRF2 (Proteintech; 16396-1-AP, 1:50) or 4-hydroxynonenal (4-HNE) (R&D Systems; MAB3249-SP, 1:100) overnight at 4 °C. Then, the sections were incubated with horseradish peroxidase-conjugated secondary antibody for 60 min at RT after washing with TBST buffer three times. Finally, the peroxidase conjugates were stained with a DAB kit (ZLI-9018, Zsbio, China), and the images were captured using an Olympus BX51 microscope (Olympus, Center Valley, PA). ImageJ was used to analyze the positive areas of IHC images. For IF staining, the HK2 cells were fixed with 4% paraformaldehyde for 15 min at RT, permeabilized and blocked with 1% BSA containing 0.1% Triton X-100 for 1 h, and labeled with primary rabbit antibody against NRF2 (Proteintech; 16396-1-AP, 1:50) overnight at 4 °C. Goat anti-rabbit secondary IgG (H+L) cross-adsorbed secondary antibody labeled with Alexa Fluor 488 (Thermo Fisher Scientific, A-11008, and 1:500) was used according to the manufacturer’s instructions. Nuclei were counterstained with DAPI (Beyotime, P0131). Images were acquired by using an LSM710 confocal microscope (Carl Zeiss, Germany). Quantification was performed using ImageJ.

**Terminal deoxynucleotidyl transferase (TdT)-mediated dUTP nick end labeling (TUNEL)**

In situ cell death was detected by using a TUNEL BrightGreen apoptosis detection kit according to the manufacturer’s instructions (A112-01/02/03, Vazyme, China). Images were captured by laser scanning confocal microscopy (LSM710, Carl Zeiss). Five random visual fields of each sample were analyzed, and the number of TUNEL-positive cells was quantified.

**Transmission electron microscopy**

Kidney tissues were fixed in 1.25% glutaraldehyde/0.1 M phosphate buffer and postfixed in 1% OsO_4_/0.1 M phosphate buffer. Ultrathin sections of 50 nm were cut on a microtome, placed onto copper grids, stained with uranyl acetate and lead citrate, and examined under an electron microscope (JEM-1010, Tokyo, Japan). Five random visual fields of each sample were analyzed, and the percentage of damaged mitochondria (characterized by swollen and disrupted cristae) was estimated as described previously ^[^[^42^](#_ENREF_42)^]^.

**Enzyme-linked immunosorbent assay (ELISA)**

Circulating levels of inflammatory factors, including IL-6 and TNF-α, were detected by ELISA kits (DAKEWEI, Shenzhen, China) according to the manufacturer's instructions.

**Assessment of lipid peroxidation by BODIPY 581/591 C11 staining**

HK2 cells treated with or without cisplatin (10 μg/ml) for 24 h were incubated with 2 μM BODIPY 581/591 C11 (D3861, Thermo Fisher Scientific) for 40 min at 37 °C following a protocol described in previous studies ^[^[^13^](#_ENREF_13)^,^ [^19^](#_ENREF_19)^]^. The stained cells were analyzed using an LSM 710 confocal microscope (Carl Zeiss) or by flow cytometry performed using a BD FACS Canto II. Stock solutions of BODIPY 581/591 C11 were prepared by dissolving 2 mg BODIPY 581/591 C11 in 100 μl dimethyl formamide.

**Measurement of malondialdehyde (MDA), GSH, and GSSG**

The levels of MDA in the kidney tissues were detected by using a lipid peroxidation MDA assay kit (Beyotime; S0131) according to the manufacturer’s instructions. A commercially available GSH and GSSG assay kit (Beyotime, China, S0053) was used to measure the levels of reduced glutathione (GSH) and oxidized glutathione disulfide (GSSG) in the kidney tissues and HK2 cells. Briefly, tissues or cultured cells were collected, washed with ice-cold PBS three times, and lysed in lysis buffer; the supernatant was collected and used for GSH and GSSG detection. The total GSH level was detected using the DTNB-GSSG cycling assay.

**Detection of cell viability and cell death**

A CCK-8 assay kit (KGA317, KeyGen Biotech, China) was used to analyze cell viability. Briefly, HK2 cells were seeded in 96-well plates and treated with MMF (2.5-200 µM) in serum-free medium for 24 h. Then, 10 μL of CCK-8 reagent was added to the medium and incubated for 1.5 h. The absorbance was measured at 450 nm using a microplate reader (Thermo Fisher, Shanghai, China). Cell death induced by cisplatin was also analyzed using an apoptosis detection kit (BD Biosciences, 556547, San Diego, CA) according to the manufacturer’s instructions. The stained cells were analyzed by flow cytometry performed using CytoFLEX (Beckman coulter). Alternatively, cell death was detected by measuring the levels of released lactate dehydrogenase (LDH) using an automatic biochemical analyzer at Children’s Hospital of Nanjing Medical University.

**Mitochondrial membrane potential detection**

The mitochondrial membrane potential (MMP) of HK2 cells was detected with tetramethylrhodamine methyl ester (TMRM, Thermo Fisher, I34361) as described in our previous study ^[^[^39^](#_ENREF_39)^]^. Briefly, HK2 cells were seeded on sterile polylysine-coated dishes and treated with cisplatin for 24 h with or without MMF. Then, the cells were incubated with TMRM in the dark for 30 min at 37 °C and washed three times with PBS. Nuclei were counterstained with Hoechst 33342, and fluorescence images were captured by confocal microscopy (LSM710, Carl Zeiss); the mean fluorescence intensity (MFI) of TMRM was analyzed by ImageJ.

**RNA sequencing**

The kidney tissues of cisplatin-induced mice treated with or without DMF were used for RNA sequencing (RNA-seq). RNA isolation, library construction, and sequencing were performed by BGI using a BGISEQ-500 RNA-seq platform (Beijing Genomic Institution, www.genomics.org.cn, BGI). The differentially expressed genes in the three groups, including vehicle, vehicle + cisplatin, and DMF + cisplatin, were defined by the BGI bioinformatics platform. All original sequence datasets have been submitted as the supplementary data (Supplementary Table S2).

**Statistical Analyses**

All data are presented as the mean ± standard deviation (S.D.). Statistical analysis was performed by unpaired Student’s t test or ANOVA using GraphPad Prism 6 (GraphPad Software, San Diego, CA). A value of P < 0.05 was considered statistically significant. Individual P values are marked in the figure legends.
